# Transcriptional response to hypoxic stress in melanoma and prognostic potential of GBE1 and BNIP3

**DOI:** 10.18632/oncotarget.22150

**Published:** 2017-10-30

**Authors:** Stéphanie Buart, Stéphane Terry, Muhammad Z. Noman, Emilie Lanoy, Céline Boutros, Paul Fogel, Philippe Dessen, Guillaume Meurice, Yann Gaston-Mathé, Philippe Vielh, Séverine Roy, Emilie Routier, Virginie Marty, Sophie Ferlicot, Luc Legrès, Morad El. Bouchtaoui, Nyam Kamsu-Kom, Jane Muret, Eric Deutsch, Alexander Eggermont, Jean-Charles Soria, Caroline Robert, Salem Chouaib

**Affiliations:** ^1^ INSERM UMR1186, Integrative Tumor Immunology and Genetic Oncology, Gustave Roussy, Equipe Labellisée par La Ligue Contre Le Cancer, EPHE, Faculté de Médecine, Université Paris-Sud, Université Paris-Saclay, Villejuif, France; ^2^ INSERM UMR 1018, Gustave Roussy, Université Paris-Sud, Université Paris-Saclay, Villejuif, France; ^3^ Department of Medical Oncology, Gustave Roussy, Villejuif, France; ^4^ Independent Consultant, Paris, France; ^5^ Plateforme de Bioinformatique, UMS AMMICA, Gustave Roussy, Villejuif, France; ^6^ YGM Consult, CEO, Paris, France; ^7^ Département de Biologie et Pathologie Médicales, Gustave Roussy, Villejuif, France; ^8^ Service d’Anatomie Pathologique, Hôpitaux Universitaires Paris Sud, AP-HP, Le Kremlin Bicêtre, France; ^9^ Laboratoire de Pathologie, INSERM UMR_S-1165/Université Paris-Diderot, Sorbonne Paris Cité, Paris, France; ^10^ INSERM UMR 981, Gustave Roussy, Université Paris-Sud, Université Paris-Saclay, Villejuif, France; ^11^ Department of Radiation Oncology, Gustave Roussy, Villejuif, France; ^12^ Drug Development Department (DITEP), Gustave Roussy, Villejuif, France; ^13^ INSERM U1030, Molecular Radiotherapy, Gustave Roussy, Université Paris-Sud, Université Paris-Saclay, Villejuif, France; ^14^ Faculty of Medicine, Université Paris-Sud, Université Paris-Saclay, Le Kremlin-Bicêtre, France

**Keywords:** hypoxia, melanoma, GBE1, glucose transporter 1, BNIP3

## Abstract

Gradients of hypoxia occur in most solid tumors and cells found in hypoxic regions are associated with the most aggressive and therapy-resistant fractions of the tumor. Despite the ubiquity and importance of hypoxia responses, little is known about the variation in the global transcriptional response to hypoxia in melanoma. Using microarray technology, whole genome gene expression profiling was first performed on established melanoma cell lines. From gene set enrichment analyses, we derived a robust 35 probes signature (hypomel for HYPOxia MELanoma) associated with hypoxia-response pathways, including 26 genes up regulated, and 9 genes down regulated. The microarray data were validated by RT-qPCR for the 35 transcripts. We then validated the signature in hypoxic zones from 8 patient specimens using laser microdissection or macrodissection of Formalin fixed-paraffin-embedded (FFPE) material, followed with RT-qPCR. Moreover, a similar hypoxia-associated gene expression profile was observed using NanoString technology to analyze RNAs from FFPE melanoma tissues of a cohort of 19 patients treated with anti-PD1. Analysis of NanoString data from validation sets using Non-Negative Matrix Factorization (NMF) analysis (26 genes up regulated in hypoxia) and dual clustering (samples and genes) further revealed that the increased level of BNIP3 (Bcl-2 adenovirus E1B 19 kDa-interacting protein 3)/GBE1 (glycogen branching enzyme1) differential pair correlates with the lack of response of melanoma patients to anti-PD1 (pembrolizumab) immunotherapy. These studies suggest that through elevated glycogenic flux and induction of autophagy, hypoxia is a critical molecular program that could be considered as a prognostic factor for melanoma.

## INTRODUCTION

Cancer immunotherapy has recently emerged as an important treatment modality. FDA approval of sipuleucel-T (Provenge^*^), ipilimumab (Yervoy^*^), nivolumab (Opdivo^*^) and pembrolizumab (Keytruda^*^) has started to deliver on the long awaited promise of cancer immunotherapy [1]. Many new modalities of immunotherapies targeting cytotoxic T lymphocytes (CTLs) responses, such as adoptive cell therapies and vaccines, are in advanced clinical trials. The ultimate goal of most cancer immunotherapy strategies is to induce a strong cytotoxic T lymphocyte (CTL) response [2]. The prevailing view is that induced or boosted CTLs will eradicate tumor cells. However, this view has been seriously challenged by clinical observations [3]. Immunotherapy effectiveness is dependent on the qualitative and/or quantitative features of the killer cells and the complexity of the genomic aberrations harbored by neoplastic cells, but is also regulated by numerous dynamic properties of the tumor microenvironment [4]. Besides tumor cells, the tumor microenvironment harbors a variety of host-derived cells. It is a complex system playing an important role in tumor development and progression [5]. It involves soluble factors and metabolic changes. Among the metabolic changes, hypoxia plays a key role in sculpting tumor microenvironment [6]. Hypoxia arises due to a combination of excessive oxygen consumption by growing tumor cells and the disorganized tumour-associated vasculature [7]. Accumulating evidence indicates that hypoxia plays an important role in tumor progression, affecting both metastatic spread and selection of cells with more aggressive phenotypes [8]. It is well established that hypoxic stress is a feature of most solid tumors and is associated with poor prognosis in several cancer types [9, 10]. In the context of tumor microenvironment, tumors impose several limitations to dampen T cell immunity as T cells, experiencing the metabolic framework of growing tumors, fail to activate distinct pathways to accomplish their functional requirements. Tumor microenvironmental hypoxia is in this regard a relevant example demonstrating how the tumor microenvironment can paralyze and neutralize T cell functions [11]. It is a negative prognostic and predictive factor owing to its multiple contributions to chemoresistance, radioresistance, angiogenesis, resistance to cell death, altered metabolism and genomic instability [12].

The master regulator of the hypoxic response is the Hypoxia-inducible factor 1 (HIF-1). Several reports have identified links between cancer outcomes and the level of HIF-1α protein [6, 12]. While incipient angiogenesis in small tumors may occur independently of hypoxia, growing tumors will at some point inevitably experience inadequate nutrient and oxygen supply [12, 13]. This deprivation triggers an angiogenic switch, which is associated with reduced sensitivity to cytotoxic and genotoxic treatment and more aggressive metastatic behavior [13]. Therefore, precise knowledge of the hypoxic state of a tumor not only provides a valuable entry point to understanding tumor progression, but also holds considerable prognostic value.

Despite the ubiquity and importance of HIF-1 response and our knowledge about the variation in the global transcriptional response to hypoxia among different cell types, little is known about gene expression signatures that might relate to melanoma development and response to treatments. It is becoming clear that HIF-1α expression alone is not a reliable marker of tumor response to hypoxia. Although treatment efficacy has been improved for patients with melanoma using checkpoints inhibitors [14], the overall 5-year survival rate is still about 50%. In fact, tumors respond very heterogeneously to this treatment and biomarkers are needed. Here, we have focused on determining transcriptional response to hypoxic stress in melanoma. RNA microarray analysis was performed to examine the effect of hypoxia on gene expression. We identified a gene expression signature, and discuss the putative prognostic and predictive potential of BNIP3 and GBE1 genes in the clinical outcome of melanoma patients treated with anti-PD1 (pembrolizumab).

## RESULTS

### Transcriptional changes associated with hypoxia in human primary melanoma cell lines

**Figure 1:**
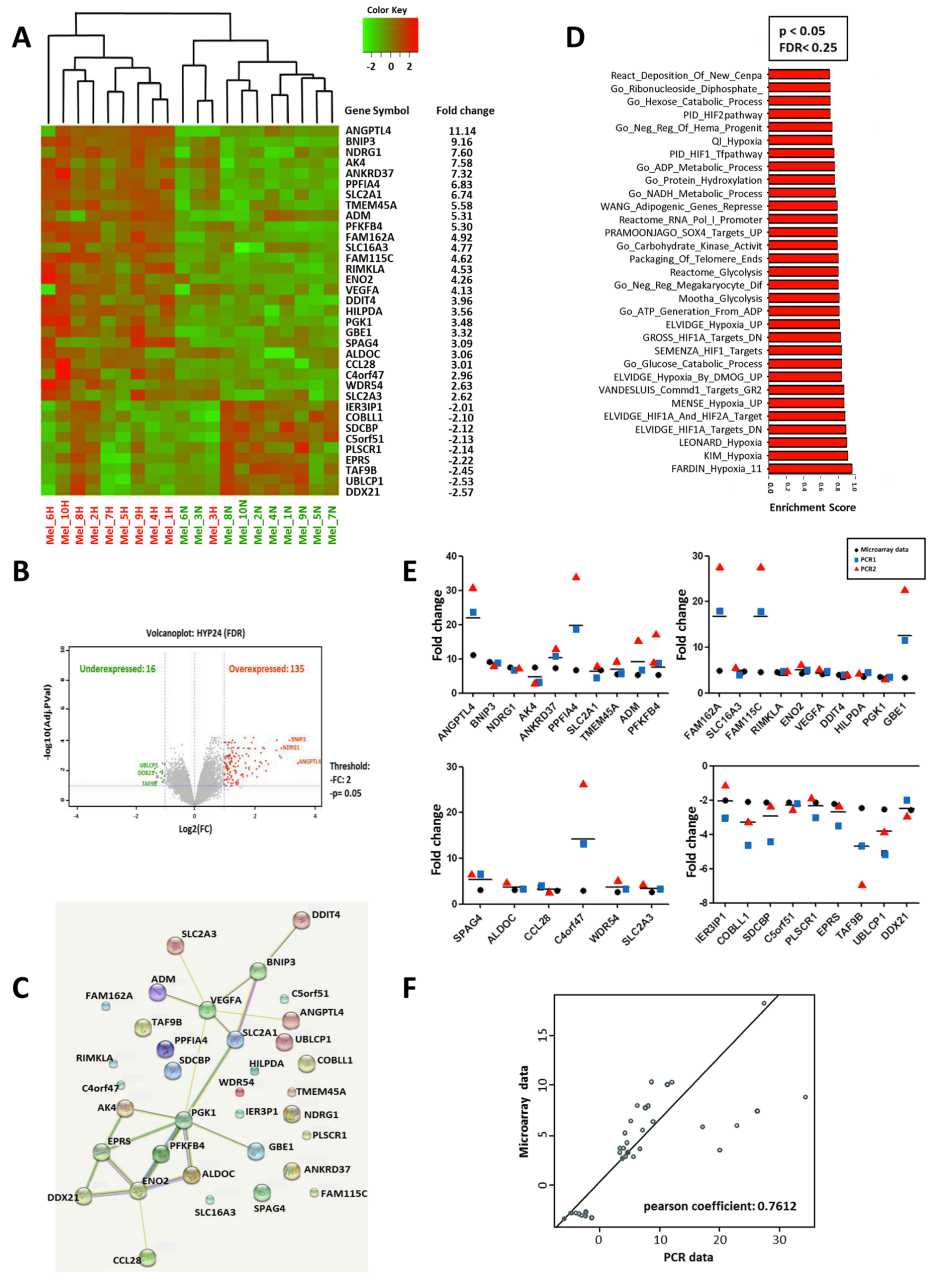
Hypoxic gene expression profiles in 10 primary human cell lines The hypomel signature was restricted at 26 genes overexpressed with fold change ≥2.50 and 9 genes underexpressed with fold change ≤-2 and p <0.005. Expression levels for individual genes were scaled by red or green color indicating an elevated or a reduced level of expression, respectively. **(A)** Heat map generated from microarray data and the gene expression fold changes of the 35 hypoxia-regulated genes in 10 primary human melanoma cell lines after 24h hypoxia (1% O2). **(B)** Volcano plot of gene expression microarray data (Log2 fold change) with adjusted p-values for the 10 primary human melanoma cell lines cultured under hypoxia 1% O2 for 24h. A total of 16 underexpressed genes and 135 overexpressed genes with a p<0.05. **(C)** STRING (Search Tool for Recurring Instances of Neighbouring Genes) approach detecting functional interactions between hypomel genes. **(D)** Graph representing the most significantly enriched gene sets/pathways (Top30) with hypoxia based on GSEA with significant FDR (FDR<0.25) and p<0.05). **(E)** Hypoxia 24h versus normoxia fold change expression for microarray data and from two independent PCR1 and PCR2 for hypomel 35 genes. **(F)** Correlation between microarray data and PCR data presented in Figure 1E: average of two independent RT-qPCR experiments (PCR1 and PCR2).

We analyzed the changes in global transcript levels in response to hypoxic stress. For this purpose, we used DNA microarrays to examine the gene expression program in response to hypoxia (1% O2) in different melanoma cell lines established from melanoma patients. We profiled global mRNA levels at the 24h time point selected on the basis of our previous finding [15]. The different mRNA samples were analyzed by hybridization to DNA microarrays. Analysis of gene expression profiles of human primary cell lines of melanoma cultured at 1% oxygen vs 21% oxygen, allowed to establish a signature of 35 genes (Figure 1A and Table 1). 26 genes up-regulated (fold-change ≥ 2.5) and 9 genes down-regulated under hypoxic conditions (fold change ≤-2) and an adjusted p-value (FDR) < 0.05 (Figure 1B). Clustering analysis clearly showed seperation of the samples into two groups according to their hypoxic status (Figure 1A). To investigate putative interactions between these genes, we then used STRING (Search Tool for Recurring Instances of Neighbouring Genes). The analysis revealed the existence of a functional interaction between 15 genes of the hypomel signature (13 genes overexpressed: BNIP3, AK4, SLC2A1, ADM, PFKFB4, ENO2, VEGFA, DDIT4, PGK1, GBE1, ALDOC, CCL28 and 2 genes underexpressed: EPRS and DDX21) (Figure 1C). A further examination of the hypomel signature using Gene Set Enrichment Analysis (GSEA) showed that several pathways were involved including hypoxia, metabolic and glucose catabolic processes (Figure 1D).

**Table 1:** List of hypoxic signature (HYPOMEL) genes

Gene Name	Gene Bank accession number	Description
*ANGPTL4*	NM_139314	Angiopoietin-like 4
BNIP3^*^	NM_004052	BCL2/adenovirus E1B 19kDa interacting protein 3
*NDRG1*	NM_006096	N-myc downstream regulated 1
AK4	NM_001005353	Adenylate kinase 4, nuclear gene encoding mitochondrial protein
*ANKRD37*	NM_181726	Ankyrin repeat domain 37
PPFIA4	NM_015053	Protein tyrosine phosphatase, receptor type, f polypeptide, interacting protein (liprin), alpha 4
SLC2A1^*^	NM_006516	Solute carrier family 2 (facilitated glucose transporter)
TMEM45A	NM_018004	Transmembrane protein 45A
ADM	NM_001124	Adrenomedullin
*PFKFB4*	NM_004567	6-phosphofructo-2-kinase/fructose-2,6-biphosphatase 4
FAM162A	NM_014367	Family with sequence similarity 162, member A
SLC16A3	NM_001042422	Solute carrier family 16, member 3
FAM115C	NM_173678	Family with sequence similarity 115, member C
RIMKLA	NM_173642	Ribosomal modification protein rimK-like family member A
ENO2	NM_001975	Enolase 2 (gamma, neuronal)
VEGFA^*^	NM_001025366	Vascular endothelial growth factor A
*DDIT4*	NM_019058	DNA-damage-inducible transcript 4
HILPDA	NM_013332	Chromosome 7 open reading frame 68
*PGK1*	NM_000291	Phosphoglycerate kinase 1
GBE1	NM_000158	Glucan (1,4-alpha-), branching enzyme 1
SPAG4	NM_003116	Sperm associated antigen 4
*ALDOC*	NM_005165	Aldolase C, fructose-bisphosphate (ALDOC), mRNA
CCL28	NM_148672	Chemokine (C-C motif) ligand 28
C4orf47	NM_001114357	Chromosome 4 open reading frame 47
WDR54	NM_032118	WD repeat domain 54
SLC2A3^*^	NM_006931	Solute carrier family 2 (facilitated glucose transporter), member 3
IER3IP1	NM_016097	Immediate early response 3 interacting protein 1
COBLL1	NM_014900	COBL-like 1
SDCBP	NM_005625	Syndecan binding protein (syntenin)
C5orf51	NM_175921	Chromosome 5 open reading frame 51
PLSCR1	NM_021105	Phospholipid scramblase 1
EPRS	NM_004446	Glutamyl-prolyl-tRNA synthetase
TAF9B	NM_015975	TATA box binding protein (TBP)-associated factor
UBLCP1	NM_145049	Ubiquitin-like domain containing CTD phosphatase 1
DDX21	NM_004728	DEAD (Asp-Glu-Ala-Asp) box polypeptide 21

The genes that overlap with other hypoxia signatures are in italic. The genes with a ^*^ are frequently up-regulated under hypoxia.

We next performed RT-qPCR analysis and confirmed the expression levels of hypoxia-associated genes identified by microarray assay (Figure 1E). An analysis of the correlation between expression level fold changes derived from microarray and RT-qPCR experiments is depicted in Figure 1F and revealed a significant correlation (Pearson’s r = 0.76; p<0.0001). Thus, the microarray data highly corroborate with those obtained by RT-qPCR for the 35 gene expression in each of the 10 tested human cell lines. Data depicted in Supplementary Figure 1 indicate that the genes associated with hypoxic stress are specifically induced in melanoma and not in peripheral blood mononuclear cells (PBMC) cultured under hypoxic conditions.

### Comparative analysis of hypoxia-associated gene expression in primary and metastatic melanoma

**Figure 2:**
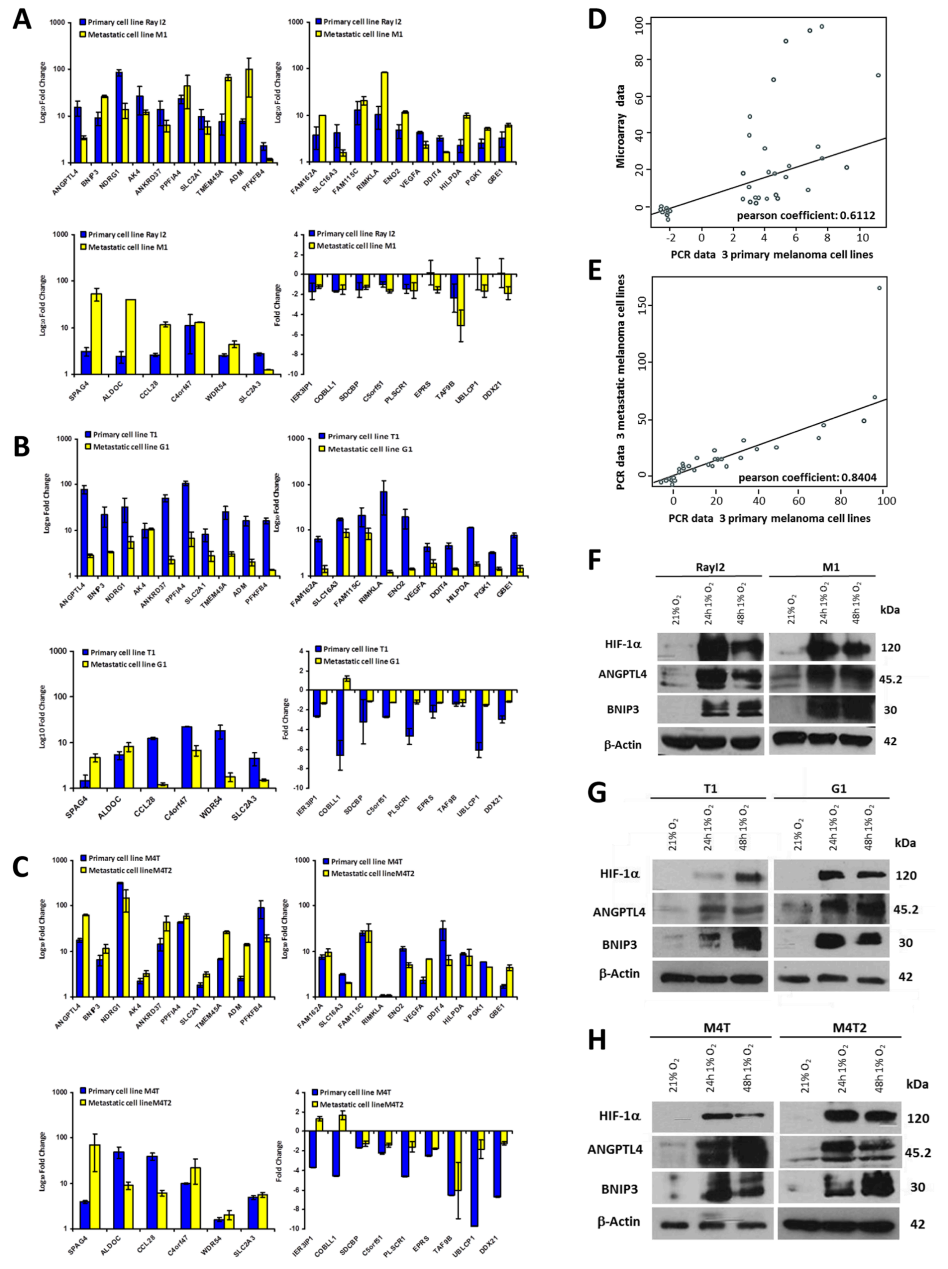
Quantification by RT-qPCR of hypomel 35 genes signature in 3 pairs of primary and metastatic cell lines from 3 patients **(A)** Patient 1 from whom were derived primary cell line Ray I2 and metastatic cell line M1. **(B)** Patient 2 from whom were derived primary cell line T1 and metastatic cell line G1. **(C)** Patient 3 from whom were derived primary cell line M4T and metastatic cell line M4T2. The 35 genes were quantified in two independent experiments after reverse transcription of total extraction mRNA from cells cultivated in normoxia and hypoxia 24h. **(D)** Correlation between microarray (from the 10 primary human melanoma cell lines) and RT-qPCR data of 3 primary cell lines (Ray I2, T1 and M4T) presented in Figure 2A, 2B and 2C. **(E)** Correlation between RT-qPCR data in A, B and C from 3 metastatic cell lines (M1, G1 and M4T2) and RT-qPCR data from 3 primary cell lines (Ray I2, T1 and M4T). Quantification by Western-blot of 3 proteins highly induced under hypoxia 16h, 24h and 48h (HIF-1α, ANGPTL4 and BNIP3) versus actin in 3 couples of primary and metastatic cell lines from 3 patients. **(F)** Patient 1 from whom were derived primary cell line Ray I2 and metastatic cell line M1. **(G)** Patient 2 from whom were derived primary cell line T1 and metastatic cell line G1. **(H)** Patient 3 from whom were derived primary cell line M4T and metastatic cell line M4T2.

We next examined the gene expression, by RT-qPCR, in response to hypoxic stress on a second set of melanoma cell lines freshly established from primary and metastatic tumors derived from 3 melanoma patients (Figure 2A, 2B and 2C). Further analysis indicates a correlation between the fold change expression levels obtained by microarray in the previous assay and the RT-qPCR analysis (Figure 2D) (Pearson’s r = 0.61; p<0.0001). Furthermore, comparative analysis of gene expression levels, as assessed by RT-qPCR, in primary vs metastatic cell lines indicates a strong correlation of gene expression between the two cell types (Figure 2E) (Pearson’s r = 0.84; p<0.0001). Using the same experimental system, Western blot analysis on two highly expressed genes (ANGPTL4 and BNIP3) confirmed their induced expression concomitant with HIF-1α induction in the cells when cultured under hypoxic conditions at 24h and 48h (Figure 2F, 2G and 2H). These results, obtained from materials freshly isolated from patient tumors, substantiate the deregulation of genes of the hypomel signature in melanoma cells. It further indicates that primary and their metastatic counterparts will respond similarly, with respect to these genes under hypoxic stress.

### Hypoxia-associated gene expression in hypoxic zones detected by anti-HIF-1α immunohistochemistry (IHC) in 8 melanoma patient tissues

**Figure 3:**
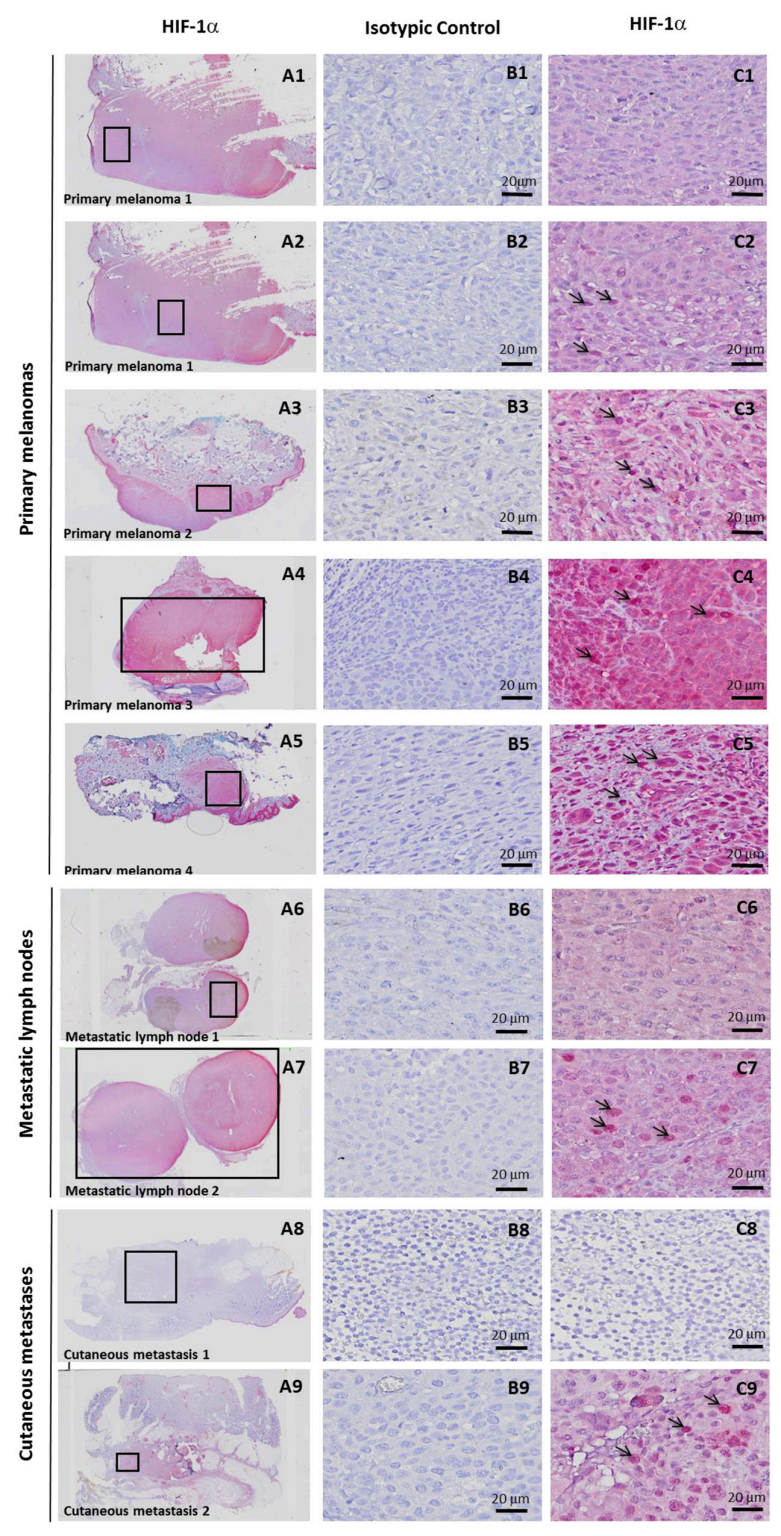
Hypoxia-associated gene expression in hypoxic zone detected by immunohistochemistry (IHC) with anti-HIF-1α in 8 melanoma cases IHC detection of HIF-1α on serial sections of FFPE tissues showing 4 hypoxic (C2, C3, C4, C5) and 1 non hypoxic (C1) zones, delimited by rectangles in A1 to A5, in primary melanoma from 4 patients. A1 and A2 is the same primary melanoma with one hypoxic zone (A2) and one non hypoxic zone (A1). Specific staining is observed in C2 (as compared with C1) in tumor nuclei (arrows). C3, C4 and C5 also show nuclear staining (arrows) with a variable cytoplasmic staining considered as non specific. IHC Identification of hypoxic (C7 and C9) and non hypoxic (C6 and C8) zones, delimited by rectangles in A6 to A9 in 2 metastatic lymph nodes (A6 and A7) and 2 cutaneous metastases (A8 and A9) from 4 patients. Magnification × 20 in A. Magnification × 400 in B and C. A1 to A9 and C1 to C9: immunostaining with anti-HIF-1α B1 to B9 : immunostaining with isotypic control.

**Figure 4:**
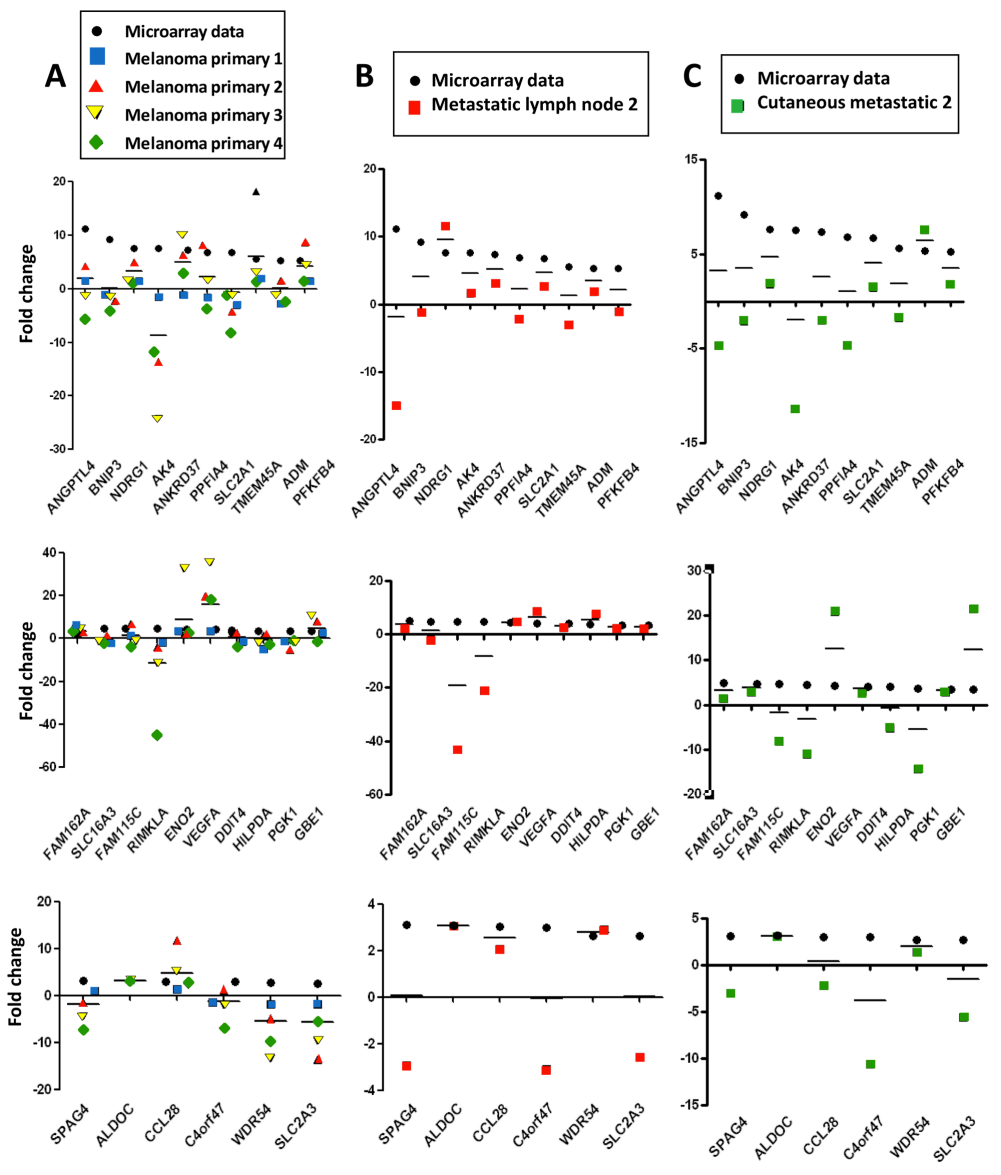
Hypomel genes expression by RT-qPCR in FFPE hypoxia zone positive after macro or microdissection from 8 tissue patients (melanoma primary, metastatic lymph node and cutaneous metastatic) Representation of data microarray fold changes (from cell lines) and PCR data fold changes derived from HIF-1α positive zone vs HIF-1α negative zone **(A)** 4 patients with primary melanomas : HIF-1α positive zone primary melanomas (zone A2, A3, A4 and A5) vs HIF-1α negative primary melanoma (zone A1). **(B)** 2 patients with metastatic lymph nodes : HIF-1α positive metastatic melanoma zone (zone A7) vs HIF-1α negative metastatic melanoma zone (zone A6). **(C)** 2 patients with cutaneous metastases : HIF-1α positive cutaneous metastase zone (zone A9) vs HIF-1α negative cutaneous metastase zone (zone A8). The average of transcript levels of PPIA, GAPDH and ACTB were used as endogenous controls genes.

We then investigated tissue specimens from patients with melanoma. Using IHC and HIF-1α on FFPE serial sections, we could identify (Figure 3) non hypoxic (A1) and hypoxic zones (A2, A3, A4, A5) in primary melanoma from 4 patient tissues. A1 and A2 are from the same primary melanoma. In this survey, It should be noted that HIF-1α positivity was considered specific only when the staining was found in cancer cell nuclei (arrows) while cytoplasmic staining was considered as non specific. Analysis of metastatic tissues illustrates hypoxic (A7 and A9) and non hypoxic (A6 and A8) zones, in 2 metastatic lymph nodes and 2 cutaneous metastases. As depicted in Figure 3, the number of HIF-1α positive cells as well as the intensity of staining can vary in the tumor zone. When the HIF-1α positive or negative zones had a limited surface, laser dissection of hypoxic or non hypoxic zone of the patient specimen was required for subsequent transcriptional analysis. To this end, we dissected the hypoxic and non hypoxic areas in melanoma samples from 8 patients who developed primary melanoma, metastatic lymph node, or cutaneous metastasis. RNAs were extracted then amplified by RT-qPCR for the 26 up-regulated genes of interest in tumor zones delimited after HIF-1α immunostaining (Figure 4A, 4B and 4C) and compared to microarray fold changes obtained in the hypomel signature derived from cell lines.

In Figure 4A, RT-qPCR data obtained were comparable for the 4 hypoxic zones of primary melanoma when normalized to a non hypoxic zone. We noted that the gene expression fold changes measured by qRT-PCR for the zones of interest (melanoma primary, metastatic lymph node and cutaneous metastatic) were similar in most instances, but often slightly reduced as compared with those obtained in the hypomel signature derived from primary cell lines.

**Figure 5:**
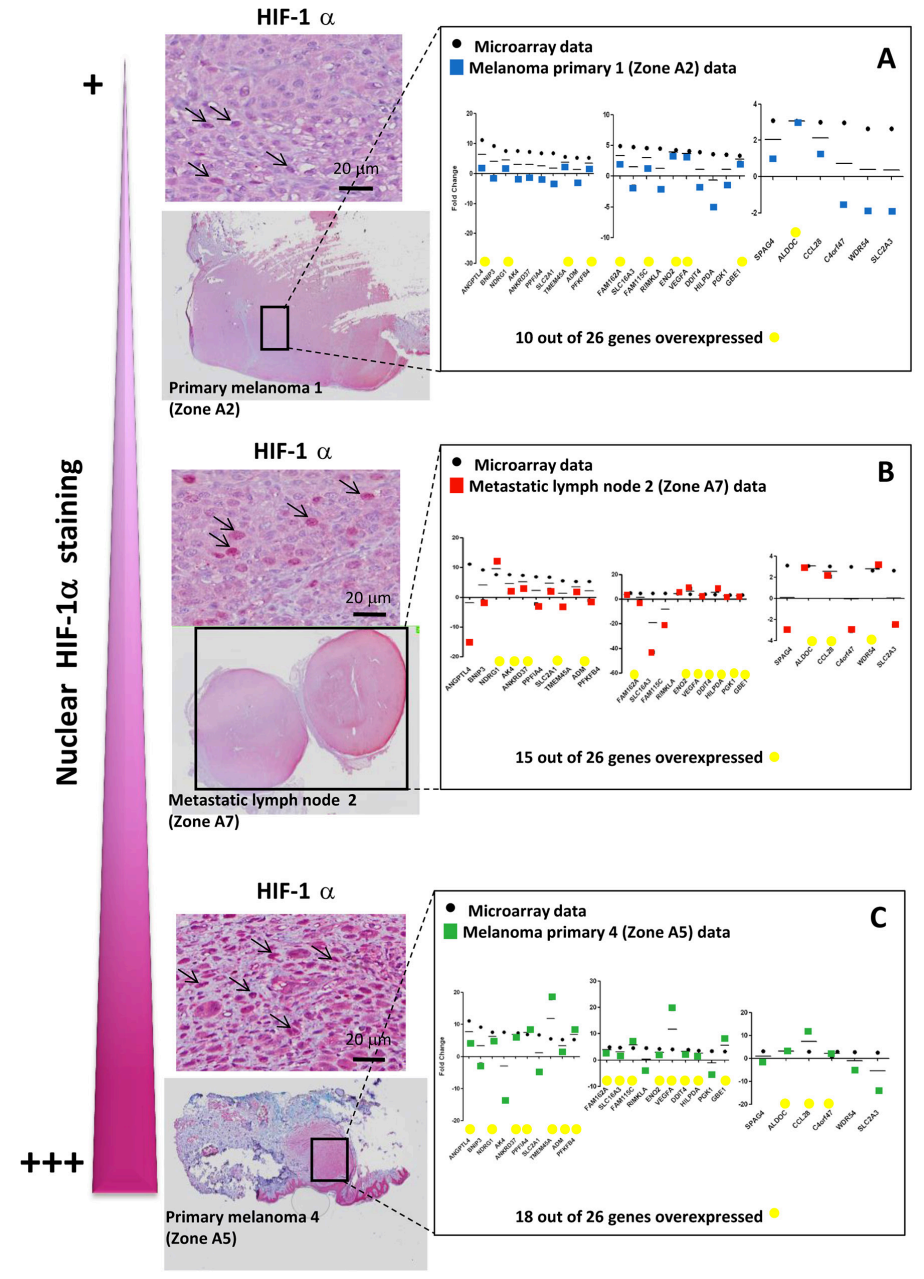
The intensity of nuclear HIF-1α staining is linked to the number of up-regulated hypomel genes **(A)** Data microarray data (from cell lines), and RT-qPCR results for HIF 1α positive primary melanoma 1 zone (zone A2) vs HIF-1α negative primary melanoma 1 zone (zone A1, as in Figure 3). **(B)** Data microarray data (from cell lines), and RT-qPCR results for HIF-1α positive metastatic lymph node 2 zone (zone A7) vs HIF-1α negative metastatic lymph node 1 (zone A6, as in Figure 3). **(C)** Data microarray data (from cell lines), and RT-qPCR results for HIF-1α positive primary melanoma 4 zone (zone A5) vs HIF-1α negative primary melanoma 1 zone (zone A1, as in Figure 3). Specific staining is observed in cancer cell nuclei (arrows). Cytoplasmic staining is considered as non specific.

This difference could be explained in part by the fact that in the different hypoxic zones, only a fraction of cells were actually imposed to hypoxic conditions (as shown by HIF-1α immunostaining), whereas in our cell line-based assays, virtually all the cells were exposed to hypoxic conditions. Moreover, by comparing HIF-1α immunostainings in patients with primary melanoma (Figure 5A and 5C) or metastatic lymph nodes (Figure 5B), we observed that tumor hypoxic zones with the strongest staining of HIF-1α were associated with a higher expression of most of the 26 up-regulated genes of the hypomel signature, suggesting a link between hypoxia, HIF-1 expression enrichment of the hypomel signature.

The recent application of the NanoString as a reliable gene expressiosn analysis prompted us to test whether the expression of our selected genes was correlated to treatment response in 19 melanoma patients treated with anti-PD1 (Supplementary Table 1). The NanoString approach offers a valuable alternative to RT-qPCR, is more accurate, and needs less materials because of direct quantification of gene copy number without the need for enzymatic amplification. We first validated this approach using 8 samples from patient tissues and cell lines with known hypoxic status. As shown in Supplementary Figure 2, a correlation exists between Nanostring data and the data previously obtained by microarray and RT-qPCR on cell lines (Pearson’s r = 0.506 ; p=0.00835), reinforcing the robustness of the NanoString analysis.

We next applied this approach for the 26 up-regulated genes within the hypomel signature using the 19 melanoma patient samples treated with anti-PD1 (9 responders (R) and 10 non responders (NR)). Finally, we investigated whether some genes within the signature could have a predictive value for the clinical outcome.

**Figure 6:**
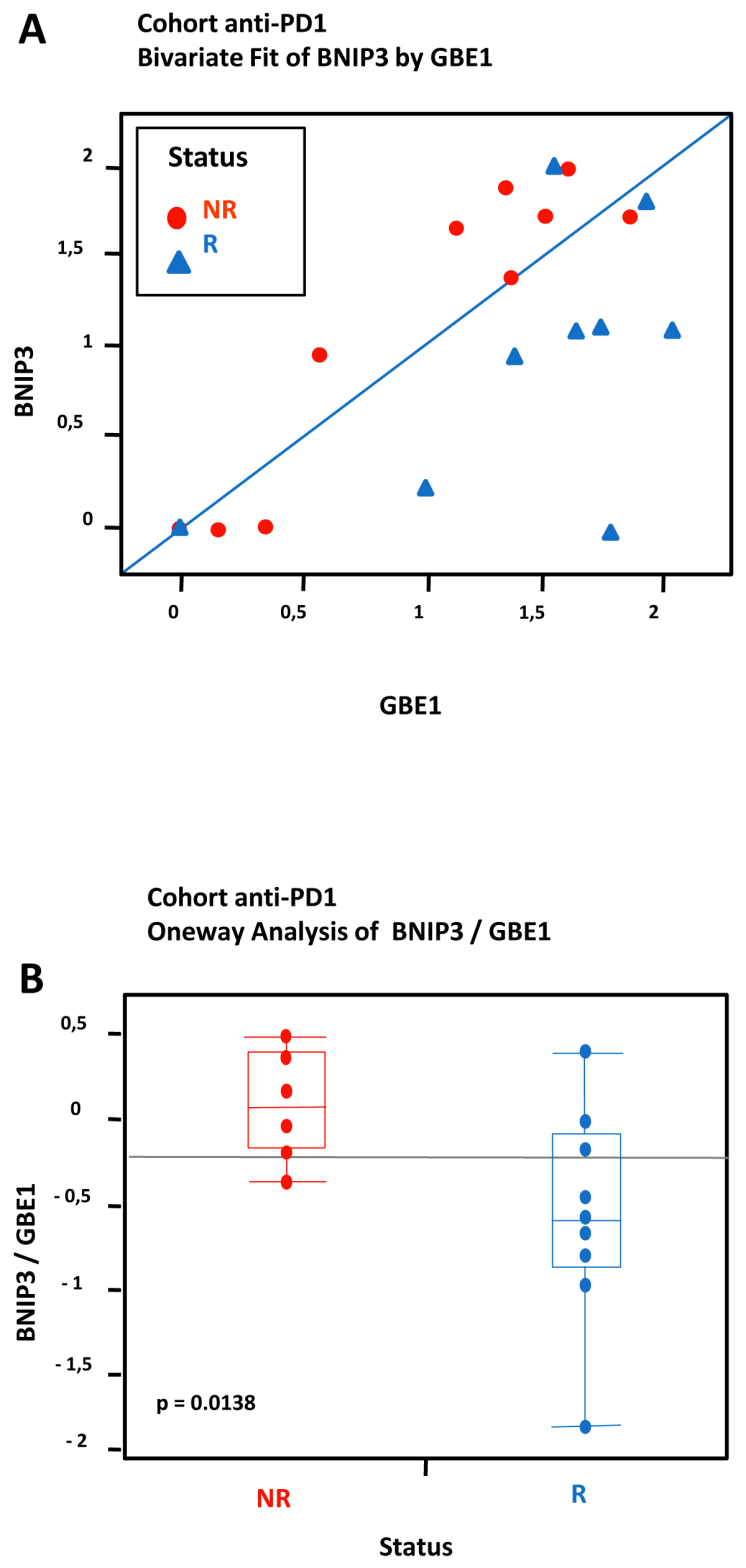
Hypoxia-associated gene expression in a cohort of 19 FFPE tumors from melanoma patients treated with anti-PD1: Data from transcriptional analysis by NanoString from 19 melanoma cases analyzed by statistical method based on differential pair analysis Correlation between expression of hypoxia genes BNIP3/GBE1 in tumors and response to treatment anti-PD1. The cohort consisted of a total of 19 patients including 9 patients who were responders (R) and 10 patients who were non responders (NR) to anti-PD1 treatment. **(A)** Most R samples appear markedly different from NR samples due to higher levels of GBE1. **(B)** A t-test demonstrates a significant difference between NR and R (log-fold (BNIP3/GBE1), p = 0.0138).

To compensate for processes unrelated directly to hypoxia, we used the “biomarker pair” approach under the rationale that such pairs would normally share common biological properties yielding similar expression levels, however could differ in their response to treatment [16]. We identified 18 pairs of highly correlated pairs (correlation level > 0.80) based on the raw expression levels of the 26 studied genes. Then, we applied Nonnegative matrix factorization analysis (NMF) to the matrix consisting of samples in (rows) and expression ratios between each selected pair of genes as variable. NMF seeks matrix factors containing only nonnegative elements. The resulting factorization often leads to substantial improvements in interpretability of the factors and in prediction of the outcome [17]. Prototypical expression patterns are estimated, in effect yielding a bi-ordering of samples and genes within clusters. In case of association with responder status, a majority of samples with a given status should be grouped together on top of a given cluster. In order to assess the significance of such grouping, a permutation test is performed, as described further in the methods section. The NMF-based ordering of samples appeared significantly associated with the responder status (p = 0.041 based on 100000 permutations). The dual ordering of genes pointed to the BNIP3/GBE1 differential pair, which had the largest leverage on the second cluster. Responder status appeared significantly associated to BNIP3/GBE1 differential expression level (p=0.0138) following a one-way analysis of the differential pair (Figure 6A and 6B).

## DISCUSSION

Tumor hypoxia is one of the most important features of the tumor microenvironment, exerting an adverse effect on tumor aggressiveness and patient prognosis. In the course of these studies, we have identified a gene-expression profile of the hypoxia response in freshly established melanoma cell lines. We established a signature (called hypomel for HYPOxia MELanoma) of 35 genes: 26 genes up-regulated (fold-change ≥ 2.5) and 9 genes down-regulated under hypoxic conditions (fold change ≤ -2). The hypomel signature was validated in tumors FFPE from patients who developed primary melanoma, metastatic lymph node, or cutaneous metastasis. We have analyzed transcriptional expression by RT-qPCR of the hypomel signature in hypoxic zones delimited after IHC staining with anti-HIF-1α. RNAs were extracted from the hypoxic zones which were levied by microdissection laser. We found that a high staining with anti-HIF-1α in tumor hypoxic zone associated with a high expression of genes belonging to the hypomel signature. Based on the evidence currently available, it now appears that both the adaptive and innate immune systems can recognize and eliminate tumors. The problem we face, however, is that the tumor microenvironment is able to neutralize and paralyze both responses. One challenge for tumor immunologists in the future, is identifying patients for who will respond favorably to immunotherapy. Accordingly, in the course of these studies, we attempted to examine whether this identified signature correlates with clinical outcome of melanoma patients treated with anti-PD1 monoclonal antibody pembrolizumab and explored the quantitative NanoString technology to investigate whether some genes within the signature could have a predictive value for response following pembrolizumab treatment of patients with advanced melanoma. More importantly, we looked for pairs of highly correlated genes based on their raw expression levels (correlation level > 0.80). Non-Negative Matrix Factorization, NMF, was applied to the split matrix [17], yielding a dual clustering of samples and genes into two clusters. Samples and genes were ordered by decreasing leverage on their respective cluster. Finally, the association between the ordering of samples and responder status was assessed through a permutation test. Specifically, responder status was permuted among patients, the association score re-calculated and compared to the original score.

We identified 18 pairs of highly correlated pairs based on the raw expression levels of the 32 studied genes corresponding to 26 up-regulated genes and 6 housekeeping genes. The dual ordering of genes pointed to the BNIP3/GBE1 differential pair, which had largest leverage. Responder status appeared significant following a one-way analysis of the differential pair. We could demonstrate that the levels of BNIP3 and GBE1 correlate with the clinical response in melanoma patients treated with anti-PD1.

Several studies have shown that autophagy constitutes a potential target for cancer therapy and that the induction of autophagy in response to therapeutics can be viewed as having a prodeath or a prosurvival role [18], contributing to drug resistance. Our very recent studies highlighted a new hypoxia-induced pathway in which NANOG activates BNIP3L expression, contributing to autophagy induction in hypoxic tumor cells and their resistance to killing by CTL [19] further suggesting a link between hypoxia-induced resistance and autophagy-related stemness. BNIP3 is a member of the BCL-2 family of proteins with reported pro-death as well as pro-autophagic and cytoprotective functions, depending on the type of stress and cellular context [20]. In line with this, increased BNIP3 levels in melanoma patients appear to be linked with poor prognosis [20]. The induction of autophagy in response to metabolic and therapeutic stresses can have a prodeath or a prosurvival role. It should be noted that autophagy can contribute to fulfilling acute metabolic needs under starvation conditions by degrading and recycling the cargos [18]. Moreover, accumulating evidence indicates that among the various metabolic adaptations used by cancer cells to adjust to the conditions imposed by the tumor microenvironment, changes in glycogen metabolism are emerging as an essential response [21]. Of note, the induction of the melanogenic pathway may lead to robust upregulation of HIF-1-dependent and independent pathways in cultured melanoma cells, suggesting a key role for melanogenesis in regulation of cellular metabolism [22, 23]. In addition several reports indicate that melanogenesis can affect disease or therapeutic outcomes [24-26], metabolism [22, 27] or immune functions [28].

Hypoxia is known to induce adaptive changes in cell metabolism that include a switch from oxidative phosphorylation to glycolysis and increased glycogen synthesis. In this context, glycogen provides a convenient glucose reservoir during energy stress, glucose deprivation or senescence [29]. In addition, cancer genomics data indicate that elevated levels of the glycogenic enzyme GBE1 are associated with poor survival in AML [30]. Consistent with these reports, our studies point to a role of elevated glycogenic flux that correlates with a poor clinical response in melanoma treated with checkpoint inhibitor anti-PD1. These studies further suggest that channeling of glucose through glycogen may promote the survival of melanoma cells under hypoxia. Through elevated glycogenic flux and induction of autophagy, hypoxia appears to be a potential critical molecular program that could be considered as a prognostic factor for melanoma.

Nordsmark and colleagues found that pretreatment tumor oxygenation is a highly significant prognostic factor for survival after primary radiotherapy applied alone or combined with chemotherapy, surgery, or radiation sensitizers in patients with locally advanced HNSCC [31]. Increased HIF-1α expression has been reported by Mouriaux F et al. to correlate with cell proliferation and vascular markers CD31 and VEGF-A in uveal melanoma [32]. However, there was no correlation found between high HIF-1α expression and patient survival However very recently, the association of HIF-1α expression with clinicopathological characteristics and overall survival (OS) of patients with OSCC was evaluated by Zhou J et al. who found an association of HIF-1α overexpression with tumor size, tumor stage, lymph node metastasis, and overall survival [33].

The current studies establish a link between hypoxia, autophagy, glucose metabolism and clinical outcome. Indeed, much remains to be learned to further dissect this relationship. Next experiments will provide more insights into the role of hypoxic stress in shaping the anti-tumor response and its impact in cancer immunotherapy.

## MATERIALS AND METHODS

### Clinical samples

Clinical samples were collected from 19 patients, 12 females and 7 males who were treated with anti-PD1 (pembrolizumab) for a primary or metastatic melanoma. All patients had given their written informed consent in accordance with the declaration of Helsinki to participate. The main characteristics of the patients and their clinical responses according to the classical RECIST1.1 evaluation criteria are reported in the Supplementary Table 1.

### Human tumor melanoma cell lines and peripheral blood mononuclear cell (PBMC)

Human melanoma cell lines ME204 AI/ER, ME260 LN/DG, T921 DUF/ALE, ME300PB, ME290mH and NA8 derived from the primary lesion, were provided by Dr Pedro Romero (Ludwig Center for Cancer Research, Lausanne, Switzerland). M74 derived from the primary lesion was established by Pr Jotereau (CRCNA, Inserm UMR892, Nantes, France). RIOUP2 was derived from the primary lesion, by the laboratory. The 3 pairs of human melanoma cell lines : T1 and G1, M4T and M4T2, RAYI2 and M1 were derived from the primary lesion and the metastatic lymph node, respectively, of 3 patients in the laboratory. All the melanoma cells were cultured in RPMI 1640 with glutamax supplemented with 10% FCS, 1% penicillin-streptomycin, 1% sodium pyruvate at 37°C in a humidified atmosphere containing 5% CO2. All culture reagents were from ThermoFisher Scientific (Waltham, MA, USA). PBMC were generated from one healthy donor and cultured in RPMI 1640 with glutamax supplemented with IL-2 (25 U/ml; Roussel-Uclaf, Romainville, France), 10% Human AB serum (Institut Jacques Boy, Reims, France), 1% penicillin-streptomycin, 1% sodium pyruvate at 37°C in a humidified atmosphere containing 5% CO2.

### Hypoxic conditioning of tumor cells

Hypoxic treatment was conducted in a hypoxia workstation (Invivo2 400, Ruskinn, UK) in a humidified atmosphere containing 5% Co2, 1% O2 and 94% N2 at 37°C (24h and 48h). Melanoma cells for RNA and protein analysis were lysed directly in the hypoxia workstation without reoxygenation.

### Microarray assay

Gene expression analysis were performed with Agilent^®^ SurePrint G3 Human GE 8x60K Microarray (Agilent Technologies, Santa Clara, CA, USA) with the following dual-color design: the test samples (Hypoxic samples) were labeled with Cy5 whereas the control samples (normoxic samples) were labeled in Cy3 using the two-color Agilent labeling kit (Low Input Quick Amp Labeling Kit 5190-2306) adapted for small amount of total RNA (100 ng total RNA per reaction). Hybridization was then performed following the manufacturer instructions. Microarray images were analysed by using Feature Extraction software version (10.7.3.1) from Agilent technologies. Defaults settings were used.

### Microarray data processing and analysis

Raw data files from Feature Extraction were imported into R with LIMMA [34], an R package from the Bioconductor project, and processed as follows: gMedianSignal and rMedianSignal data were imported, controls probes were systematically removed, and flagged probes (gIsSaturated, gIsFeatpopnOL, gIsFeatNonUnifOL, rIsSaturated, rIsFeatpopnOL, rIsFeatNonUnifOL) were set to NA. Intra-array normalization was performed by a loess normalization, followed by a quantile normalization of both Cy3 and Cy5 channel. Then inter-array normalization was performed by quantile normalization on M values. To get a single value for each transcript, taking the mean of each replicated probes summarized data. Missing values were inferred using KNN algorithm from the package ‘impute’ from R bioconductor. Normalized data were then analyzed. To assess differentially expressed genes between two groups, we start by fitting a linear model to the data. Then we used an empirical Bayes method to moderate the standard errors of the estimated log-fold changes. The top-ranked genes were selected with the following criteria: an absolute fold-change ≥ 2.5 and ≤-2 and an adjusted p-value (FDR) < 0.005. To interogate interactions between hypomel genes and pathways, we used STRING (Search Tool for Recurring Instances of Neighbouring Genes). Gene Set Enrichment Analysis (GSEA) used the Molecular Signatures Database (MSigDB).

### RNA isolation and real-time quantitative polymerase chain reaction (RT-qPCR)

Total RNAs were extracted from cell samples using TRIzol solution (Invitrogen). The quality of RNAs was assessed using a Bioanalyzer instrument (Agilent) and then quantified using a Biospecnano (Shimadzu, Kyoto, Japan). cDNA synthesis was prepared from 1 μg of total RNA with random hexamers using Applied Biosytems Reverse Transcription kit according to the supplied protocols. Gene expression was quantified by SYBR Green qPCR method using the Maxima™ SYBR Green/ ROX qPCR Master Mix on an StepOnePlus Real Time PCR system (ThermoFisher Scientific). Relative expression was calculated by using the comparative Ct method (2-ΔΔCt). Primer sequences for the quantification of 35 genes were purchased from Sigma and are available upon request. Transcript levels of HPRT for PBMC, or 18S for melanoma cells lines were used as endogenous control.

### Western blot

Melanoma cells lines from 3 patients were grown in two different conditions normoxia (21% PO2) and hypoxia (1% PO2) for 24h and 48h at 37°C. Cancer cells were washed twice in phosphate-buffered saline and lysed in plates with lysis buffer (62.5 mM Tris-HCl [pH 6.8], 2% weight/volume sodium dodecyl sulfate, 10% glycerol, 1 mM orthovanadate, 2 mM phenylmethylsulfonyl fluoride, 25 μM leupeptin, 5 mM benzamidine, 1μM pepstatin, and 25 μM aprotinin). Lysates were sonicated on ice, resolved by sodium dodecyl sulfate-polyacrylamide gel electrophoresis (30μg/well), and transferred onto nitrocellulose membranes. The membranes were blocked in blocking buffer then probed overnight at 4°C with the indicated primary Abs. Primary antibodies (Abs) against HIF-1α (mouse Ab, Clone 54/HIF-1α 610959), BNIP3 (mouse Ab, clone ANa40, 10433), ANGPTL4 (Rabbit polyclonal Ab, SAB1410901) and β-Actin (mouse Ab, clone AC-15) were purchased respectively from BD Biosciences (San Jose, CA, USA), Abcam (Cambridge, UK) and Sigma-Aldrich (St Louis, MO, USA). The labeling was performed following incubation with horseradish peroxidase (HRP)-conjugated secondary Abs: a HRP goat anti-mouse for HIF-1α and BNIP3, and a HRP goat anti-rabbit for ANGPTL4, and detection with an enhanced chemiluminescence kit (GE Healthcare, Chicago, IL, USA). Blots were scanned and processed by Adobe Photoshop 7.0 software.

### Immunohistochemistry staining for HIF-1α

The tissue collection was composed of 4 human melanoma primary tumors, 2 human cutaneous metastasis tumors and 2 human melanoma lymph node metastases.

For each patient, four micrometer sections of Fixed-formaldehyde paraffin embedded (FFPE) melanoma human tumors were prepared and stained with HES (Hematoxylin Eosin Safran). Deparaffinized tissue sections were treated with Antigen Retrieval Solution (citrate buffer, pH 8.0, concentrated 10×, T0010 (Diapath, Martinengo, Italy) in water bath at 95°C. Tissue sections were then incubated with H2O2 3% for 10 min and solution PowerVision IHC/ISH Super Blocking PV6122 (MM France, Brignais, France) for 20 min. Histological slides were incubated over night at 4°C with a polyclonal rabbit anti-human HIF-1α antibody NB100-479 (Novus Biologicals, Littleton, CO, USA) or a rabbit polyclonal IgG Ab 27472 (Abcam). For signal amplification, slides were then incubated with rabbit alkaline phosphatase conjugated secondary antibody PowerVision poly-AP anti-Rabbit IgG PV3133 (MM France). The signal was revealed with the Liquid Permanent Red K0640 (Dako, Les Ulis, France) and Mayer’s hemalun solution counterstain (Merck Millipore, Billerica, MA, USA). In this analysis, was considered as positive nuclear immunostaining of HIF-1α in cancer cells, whereas cytoplasmic immunostaining was considered as non-specific.

### Laser microdissection and pressure catapulting of glomerules

Laser microdissection was performed with a PALM^®^ RoboSoftware 4.6 MicroBeam system (PALM Microlaser Technologies, Zeiss Micro-Imaging, Munich, Germany) coupled to an inverted microscope Axio Observer.Z1. Serial 20 μm-thick sections from 3 human melanoma FFPE fragments (primary melanoma 1, 2 and 4, Figure 3) were spread onto polyethylene naphthalate (PEN) membrane-coated slides (Carl Zeiss Micro Imaging, Munich, Germany). After sectioning, the slides were incubated for 10 min in a toluene solution followed by 10 min in absolute alcohol to completely remove the paraffin embedding. Staining for 1 min in a Mayer’s Hematoxylin solution, 30 s in a saturated lithium carbonate solution and 30 s in a solution of erythrosine were successively done followed by 30 s each of absolute alcohol and toluene, respectively.

A total surface of 4 areas (between 6.6 × 106 μm^2^ and 18 × 106 μm^2^) was collected from several sections representing the 3 identified hypoxic tumoral areas (zone A2, A3 and A5, Figure 3) and 1 non hypoxic tumoral area (zone A1, Figure 3). The total of surface was determined by the computer during microdissection process. All the surfaces were computed and the total surface was determined accordingly. Each sample was recovered in lysis buffer from the RNeasy FFPE kit (Qiagen, Hilden, Germany) for further molecular analyses.

### Macrodissection with scalpel

Serial 20 μm-thick sections from 5 human melanoma FFPE tumors (primary melanoma 4, metastatic lymph node 1 and 2, cutaneous metastasis 1 and 2, Figure 3) were prepared as samples obtained by laser microdissection. A total of 5 areas was collected with a scalpel from several sections representing the 3 identified hypoxic tumoral areas (zone A4, A7, and A9, Figure 3) and 2 non hypoxic tumoral areas (zone A6 and A8, Figure 3). Each sample was recovered in lysis buffer from the RNeasy FFPE kit (Qiagen,) for further molecular analyses.

### Pathway specific gene expression profiling of hypoxic and non hypoxic tumoral FFPE samples

Total RNAs were extracted from 9 area melanoma FFPE tissue sections obtained by laser microdissection or macrodissection with scalpel (zone A1 to A9, Figure 3), using RNeasy FFPE kit (Qiagen, Hilden, Germany). RNA quantity and quality was assessed using the Nanodrop-ND-1000 (Nanodrop Technologies, Wilmington, USA). First-strand cDNA was synthesized using a High-Capacity cDNA Reverse Transcription Kit (ThermoFisher Scientific) according to the manufacturer’s protocol. Signaling pathways transcript analyses were conducted in duplicates using a personalised Human qPCR SignArrays^®^ 384 system (gene profiling analysis Human qPCR SignArrays^®^ 384 kit for 26 genes of interest; and Perfect MasterMix SYBR Green (AnyGenes, Paris, France)) on a LightCycler 480 (Roche, Rosny-sous-Bois, France) as described by the manufacturer, in 9 FFPE samples. Quality control of qPCR data for consequent analysis was based on positive and negative PCR controls. Briefly, a total volume of 20μl of PCR mix, which included 10μl of Perfect MasterMix SYBR Green^®^, 8μl of PCR grade water and 2μl of cDNA was loaded into each well of the qPCR array. PCR amplification was conducted at 95°C for 10 min, followed by 40 cycles of 95°C for 10 sec and 60°C for 30 sec. The mRNA expression for each gene was normalized using the average expression of 3 housekeeping genes: peptidylprolyl isomerase A (cyclophilin A, PPIA), b-actin (ACTB), and Glyceraldehyde-3-phosphate deshydrogenase (GAPDH). Data analysis was conducted using AnyGenes^®^ Excel analysis tools based on ΔΔCt method by calculating fold changes for each gene as the difference in gene expression between positive HIF-1α and negative HIF-1α samples.

### Nanostring analysis

Total RNAs were extracted from a cohort of 19 tissues patients (Supplementary Table 1). 8 RNA samples already analyzed by microarray and/or by RT-qPCR were included as controls in the analysis : 6 RNA samples from 3 cell lines (Mel-1, Mel-6 and Mel-10) cultured under normoxia and hypoxia, 1 RNA sample extracted from hypoxia FFPE zone positive of a primary melanoma (zone A4, Figure 3) and 1 RNA sample extracted from hypoxia FFPE zone negative of a primary melanoma (zone A1, Figure 3). Total RNA concentration and purity (Ratio 260/280 and ratio 260/230nm) were calculated using a Nanodrop ND8000 spectrophotometer (Ozyme, Saint-Quentin en Yvelines, France). Total RNA integrity was assessed using a micro electropheresys (RNA6000 LabChip, Agilent technologies), and RIN or percentage of fragment longer than 300 nt were calculated, upon a total RNA migration. Direct quantification of mRNA was achieved according a Nanostring Custom Elements approach. 50 ng of total RNA were used as template to detect 32 targets corresponding to 26 mRNA of interest and 6 housekeeping genes. The Nanostring nSolver software was used to control raw data, and to normalize data based on geometric mean of positive controls, and water signals to deduce unspecific counts.

With the goal of identifing biomarker of response to therapy, we looked for pairs of highly correlated genes based on their raw expression levels (correlation level > 0.80), under the rationale that such correlated genes would normally share common biological properties yielding similar expression levels, however could differ in their response to treatment [16]. Within each pair of correlated genes, the difference between genes, called “differential pair”, was calculated, thereby ensuring that the variation in expression, not caused by the response to treatment itself, will be optimally filtered out. The matrix of differential pairs was further split into two parts corresponding to positive and negative differences. Within first part, negative differences were replaced by 0. Within second part, positive differences were replaced by 0, whereas negative differences were replaced by their absolute value. Non-Negative Matrix Factorization (NMF) was applied to the split matrix [17]. Specifically, two expression profiles among genes, each one linked to a particular expression pattern, were simultaneously estimated. A dual clustering of samples and genes into two clusters was achieved by grouping together samples and genes that loaded most on a particular pattern or expression profile. Finally, within each cluster, samples and genes were ordered by descending leverage on their respective cluster. The association between the ordering of samples and responder status was assessed through a permutation test. Responder status was permuted among patients and the rank-sum score was re-calculated and compared to the original score.

## SUPPLEMENTARY MATERIALS FIGURES AND TABLE



## Abbreviations

HIF: hypoxia inducible factor
