# Supplementary material for: Transcriptional response to hypoxic stress in melanoma and prognostic potential of GBE1 and BNIP3
Source: Oncotarget. 2017 Oct 30;8(65):108786–801. doi: 10.18632/oncotarget.22150 (PMC5752481; doi:10.18632/oncotarget.22150)
Supplement: Supplementary file 1 [file oncotarget-08-108786-s001.pdf]

Transcriptional response to hypoxic stress in melanoma and prognostic potential of GBE1 and BNIP3

SUPPLEMENTARY MATERIALS

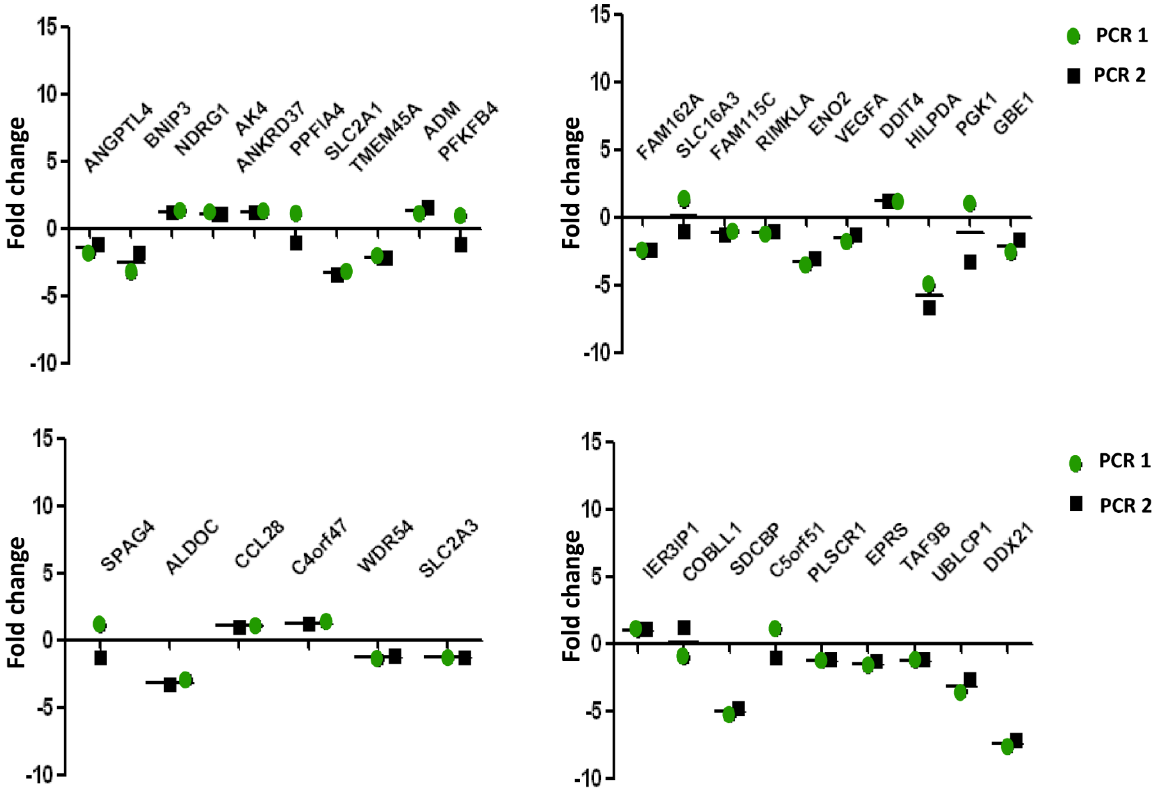

Supplementary Figure 1: Expression of Hypomel genes in PBMC evaluated by RT-qPCR. Representation of hypoxia 24h versus normoxia fold change expression for two independent PCR experiments (PCR1 and PCR2) for hypomel 35 genes in PBMC. The transcript level of HPRT was used as endogenous control.

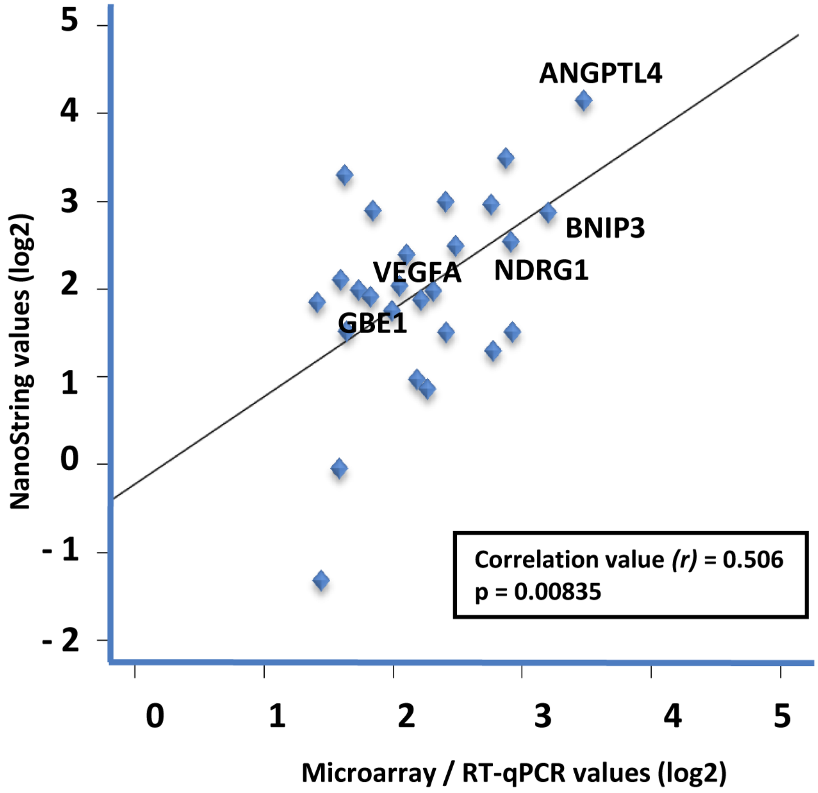

Supplementary Figure 2: Validation of robustness of NanoString technology. Comparison of gene expression values generated by microarray and NanoString for a total of 8 RNA samples with known hypoxia status including 6 RNA samples extracted from 3 cell lines cultivated in normoxia and hypoxia (Mel_1, Mel_6 and Mel_10 already used to generate microrarray data), 1 RNA sample extracted from FFPE HIF-1α positive zone of primary melanoma 3 (zone A4, Figure 3) already analyzed by RT-qPCR, and 1 RNA sample extracted from FFPE HIF-1α negative zone of primary melanoma 1 (zone A1, Figure 3) already analyzed by RT-qPCR. Correlation of gene expression values for the 8 samples (correlation value (r)=0.506, p=0.00835).

Supplementary Table 1: Clinical data of the cohort of 19 melanoma patients treated with anti-PD1. 9 patients responders (R) to treatment (noted 1R to 9R), and 10 patients non responders (NR) (noted 10NR to 19NR)

Patients	Gender[1]	Age	Treatment	Response[2]	Tumor Site	Tumor stage	TNM	Mutation
1R	F	72	Pembrolizumab	CR	sub-cutaneous	IV	TxN2cM1b	BRAF wildtype
2R	F	62	Pembrolizumab	PR	cutaneous	IIIC	T4bN2cM0	BRAF-CKIT neg
3R	M	79	Pembrolizumab	CR	lymph nodes	IV	T2aN3M1a	BRAF-NRAS-CKIT neg
4R	M	64	Pembrolizumab	CR	cutaneous	IV	T3bN3M1a	BRAF wildtype
5R	F	73	Pembrolizumab	CR	primitive melanoma	IV	T3aN3M1a	BRAF V600E+
6R	F	54	Pembrolizumab	CR	cutaneous	IV	T3bN2aM1c	BRAF V600E+
7R	M	55	Pembrolizumab	CR	cutaneous	IV	T4xN+M1c	BRAF wildtype
8R	F	32	Pembrolizumab	CR	cutaneous	IV	T1aN0M1a	BRAF V600E+
9R	F	55	Pembrolizumab	CR	cutaneous	IIIC	TxN3M0	BRAF-CKIT neg, NRAS positive Q61K
10NR	F	78	Pembrolizumab	PD	primitive melanoma (anal)	IV	T4N2bM1a	BRAF-NRAS-CKIT neg
11NR	F	53	Pembrolizumab	PD	sub-cutaneous	IV	TxN3M1b	BRAF-NRAS-CKIT neg
12NR	M	78	Pembrolizumab	PD	lymph nodes	IV	T4bN3M1c	BRAF-CKIT neg
13NR	F	66	Pembrolizumab	PD	lymph nodes	IIIC	T2aN3M0	BRAF-CKIT neg
14NR	F	53	Pembrolizumab	PD	cutaneous	IV	TxN3M1b	BRAF-NRAS-CKIT neg
15NR	M	58	Pembrolizumab	PD	primitive melanoma (skin)	IV	T4xN+M1c	BRAF V600E+
16NR	M	85	Pembrolizumab	PD	cutaneous	IV	TxN3M1c	BRAF V600E+
17NR	F	65	Pembrolizumab	PD	mucosal (vaginal)	IV	T4bN3M1c	BRAF-NRAS-CKIT neg
18NR	M	71	Pembrolizumab	PD	lymph nodes	IV	T4bN2aM1c	BRAF-CKIT neg
19NR	F	71	Pembrolizumab	PD	lung	IV	TxNxM1c	BRAF-CKIT neg

[1]M: Male, F: Female

[2]CR : Complete response, PR: Partial response and PD: Progressive disease
